# EEG Correlates of Self-Referential Processing

**DOI:** 10.3389/fnhum.2013.00264

**Published:** 2013-06-06

**Authors:** Gennady G. Knyazev

**Affiliations:** ^1^Institute of Physiology, Siberian Branch of Russian Academy of Medical Sciences, Novosibirsk, Russia

**Keywords:** self-referential processing, default mode network, EEG, ERP, oscillations

## Abstract

Self-referential processing has been principally investigated using functional magnetic resonance imaging (fMRI). However, understanding of the brain functioning is not possible without careful comparison of the evidence coming from different methodological domains. This paper aims to review electroencephalographic (EEG) studies of self-referential processing and to evaluate how they correspond, complement, or contradict the existing fMRI evidence. There are potentially two approaches to the study of EEG correlates of self-referential processing. Firstly, because simultaneous registration of EEG and fMRI has become possible, the degree of overlap between these two signals in brain regions related to self-referential processing could be determined. Second and more direct approach would be the study of EEG correlates of self-referential processing *per se*. In this review, I discuss studies, which employed both these approaches and show that in line with fMRI evidence, EEG correlates of self-referential processing are most frequently found in brain regions overlapping with the default network, particularly in the medial prefrontal cortex. In the time domain, the discrimination of self- and others-related information is mostly associated with the P300 ERP component, but sometimes is observed even earlier. In the frequency domain, different frequency oscillations have been shown to contribute to self-referential processing, with spontaneous self-referential mentation being mostly associated with the alpha frequency band.

Self-referential processing has been principally investigated using functional magnetic resonance imaging (fMRI) and positron emission tomography (PET), which currently dominate the field of human neuroscience. Electroencephalographic (EEG) studies are less numerous and, to the best of my knowledge, have not been systematically reviewed. Understanding of the brain functioning is not possible without careful comparison of the evidence coming from different methodological domains. Ideally, different methods are expected to complement each other. For example, excellent spatial resolution of fMRI could be complemented by excellent temporal resolution of EEG. In reality, however, different methods may give contradicting results. In such a case, a careful analysis of possible causes of the discrepancy is necessary. In this paper, I aimed to review EEG studies of self-referential processing and to evaluate how they correspond, complement, or contradict to the existing fMRI evidence. It is important to keep in mind that fMRI and EEG represent different aspects of brain activity and there may be a degree of incongruence between hemodynamic and electrophysiological signals. The relationship between EEG signal and concurrent changes in neuronal spiking and local field potentials are relatively well understood (e.g., Buzsaki and Draguhn, 2004; Basar, 2008). On the other hand, it is not yet clear how the changes in the blood oxygen level dependent (BOLD) signal relate to concurrent changes in neuronal events (Huettel et al., 2004; Debener et al., 2006). The quest to elucidate how the self is processed in the brain requires a solid understanding of the link between neuroimaging findings and their electrophysiological underpinnings.

Reliability and validity of a particular method is also a very important issue. Reliability is the cornerstone of any scientific enterprise. If a measurement is unreliable, it cannot be valid. However, if a method is reliable it can also be invalid (Carmines and Zeller, 1979). In this review, it is not possible to cover the issue of reliability and validity of EEG and fMRI methods in detail (for recent reviews, see e.g., Bennett and Miller, 2010; Thatcher, 2010). High levels of reliability (i.e., >0.95) of several quantitative EEG measures have been shown in many studies (e.g., Lund et al., 1995; McEvoy et al., 2000; Corsi-Cabrera et al., 2007; Gudmundsson et al., 2007; Näpflin et al., 2008; Towers and Allen, 2009; Schmidt et al., 2012). Somewhat smaller reliabilities are usually found for event-related potential (ERP) components. Thus, test-retest correlation coefficients for oddball task P300 amplitude range from 0.50 to 0.80 and for peak latency from 0.40 to 0.70 (Polich, 1986; Fabiani et al., 1987; Segalowitz and Barnes, 1993; Walhovd and Fjell, 2002). Hall et al. (2006) found higher test-retest reliability for the P300 amplitude (0.86) and latency (0.88). Less evidence exists regarding reliability of fMRI measures. Vul et al. (2009), summarizing several studies, conclude that fMRI measures computed at the voxel level will not often have reliabilities greater than about 0.7. Lieberman et al. (2009) argued that fMRI reliability was likely around 0.90. Friedman et al. (2008) show that for median percent signal change measure, the median test-retest reliability was 0.76. Aron et al. (2006) found 1-year test-retest fMRI reliability in a classification-learning task exceeding 0.8. Similar test-retest reliability of fMRI activation during prosaccades and antisaccades at the group level was shown by Raemaekers et al. (2007). However, these authors showed that reliable results could be obtained in some but not all subjects, mostly due to individual differences in the global temporal signal to noise ratio (SNR). Comprehensive discussion of the reliability of fMRI and effects of SNR could be found in Bennett and Miller (2010). Thus, it could be summarized that test-retest reliability, at least for some EEG measures, tends to be excellent and is at the border between good and excellent for most fMRI studies.

## Self-Referential Processing and the Default Mode Network

The concept of the default mode network (DMN) was first introduced by Raichle et al. (2001) basing on the evidence showing a consistent pattern of deactivation across a network of brain regions that occurs during the initiation of task-related activity (Raichle et al., 2001; Raichle and Snyder, 2007). The DMN includes the precuneus/posterior cingulate cortex (p/PCC), the medial prefrontal cortex (MPFC), and medial, lateral, and inferior parietal cortex. This network is active in the resting brain with a high degree of functional connectivity (FC) between regions. The more demanding the task the stronger the deactivation appears to be (McKiernan et al., 2006; Singh and Fawcett, 2008). A notable exception to this general pattern of deactivation during goal-directed activity occurs in relation to tasks requiring self-referential thought and social cognition (Mitchell, 2006; Gobbini et al., 2007), which suggests that the DMN likely mediates active cognitive processes rather than being strictly a “default” network, which only shows inactivation. Recent studies show that these processes include first-person perspective (Greicius et al., 2003; Vogeley et al., 2004), task-independent thoughts (Binder et al., 1999; McKiernan et al., 2003), episodic memory (Greicius and Menon, 2004), social cognition and sense of agency processes (Decety and Sommerville, 2003; Gallagher and Frith, 2003), distinction between self- and non-self-related stimuli (see Northoff et al., 2006; Buckner et al., 2008; for a review), and social interaction tasks (Rilling et al., 2004, 2008). All this evidence implies that the DMN appears to be the seat of self-referential processing in the brain.

## Approaches to the Study of EEG Correlates of Self-Referential Processes

Electroencephalogram and fMRI represent different aspects of brain activity. Moreover, different EEG measures may also relate to different aspects of neuronal activity and show little or no correlation with each other. Therefore, a brief description of most popular measures that are used in EEG domain seems necessary for clearer understanding of later discussed studies. Firstly, EEG measures could be obtained in a resting condition or during performance of different tasks or presentation of different stimuli. In the former case they represent “spontaneous” or ongoing electrical activity and could be used to investigate EEG correlates of spontaneous self-referential processes, such as mind wondering and task-unrelated-thoughts. In the latter case, different measures of event-related changes in electrical activity, such as ERP and event-related oscillations, are used to study the processing of external self-related information.

Event-related potential is a powerful and very popular tool for the study of cortical dynamics that are phase-locked to (mostly) external stimuli and events. By calculating the mean of EEG epochs, the activity phase-locked to the stimulus is preserved, whereas non-phase-locked activity cancels itself out. It should be borne in mind that ERP is not the only kind of electrical cortical responses. A portion of these responses is time-locked to the stimulus, but is not temporally synchronized with it, meaning that this activity will cancel itself out during averaging. This kind of responses is usually labeled induced responses, as distinct from evoked responses that are phase-locked to the stimulus. There has been a long debate about how ERPs are related to ongoing oscillations and induced responses (e.g., Kolev and Yordanova, 1997; Makeig et al., 2002; Jansen et al., 2003; Klimesch et al., 2004). Most researchers agree that evoked and induced responses represent different aspects of brain function. Much evidence shows that evoked responses (e.g., different ERP components) are involved in stimulus perception and processing, that is, bottom-up processes. Induced responses, on the other hand, do not probably directly participate in stimulus perception and processing. However, they are involved in concomitant top-down processes, such as allocation of attention, memory retrieval, decision-making, and emotion. Linking evoked responses with bottom-up and induced responses with top-down processes is consistent with the theoretical framework suggested by David et al. (2006) who associate evoked and induced responses with “drivers” and “modulators,” respectively. The mechanisms of action of drivers refer to classical neuronal transmission, either biochemical or electrical. Modulatory effects can engage a complex cascade of highly non-linear cellular mechanisms (David et al., 2006).

Oscillations are the most salient feature of EEG. They could be studied both in rest and during processing of external stimuli or tasks. Ongoing and event-related oscillations are usually categorized into five frequency bands: delta (0.5–3.5 Hz), theta (4–7 Hz), alpha (8–12 Hz), beta (13–30 Hz), and gamma (>30 Hz), although there is generally a lack of consistency between studies with maintaining a standard range of EEG bands. The five major bands are frequently subdivided into narrower sub-bands and there is no general agreement as to the boundaries of these sub-bands. This is a potential source of discrepancies in results of different studies. It was also suggested that there are substantial individual differences in EEG frequency band boundaries and they should be individually adjusted using alpha peak frequency as the anchor (Klimesch, 1999). These debates have partly lost their actuality due to the advent of modern methods of time-frequency representation, such as wavelet transform, and adoption of mass-univariate statistical approaches (e.g., Delorme and Makeig, 2004).

It is increasingly becoming clear that oscillations may have a special and very important role in the integration of brain functions (Nunez, 2000; Varela et al., 2001; Cantero and Atienza, 2005; Palva et al., 2005; Knyazev, 2007; Basar, 2008; Fingelkurts and Fingelkurts, 2010). Two different aspects of EEG oscillations could be potentially measured: the power of a particular oscillation at different cortical locations and its synchrony (i.e., phase consistency) over these locations. The former is usually measured by means of different time-frequency transforms, such as Fourier or wavelet transform, the latter by means of coherence or similar measures. To evaluate event-related changes in oscillatory activity EEG is usually recorded before (the baseline) and during (the test period) presentation of stimuli or performance of a task; EEG changes in the test period relative to baseline are treated as “event-related” activity and are believed to reflect brain activation involved in the processing of the task in hand. Event-related oscillations are subdivided into evoked (phase-locked to the stimulus) and induced (non-phase-locked to the stimulus) parts, the latter usually being much larger in amplitude than the former. According to the currently most popular theory, the former oscillations are the building blocks of the ERP (e.g., Makeig et al., 2002; Klimesch et al., 2004). Beyond ERPs and oscillations, the global “microstates” (i.e., quasistable and unique topographic distributions of the whole-cortex electrical field potential, Lehmann, 1990) and local “microstates” (i.e., quasistable states within individual cortex locations, Fingelkurts and Fingelkurts, 2010) could be investigated both in rest and during performance of tasks.

Spatial localization of observed effects is an important and rather complicated issue in EEG research. Scalp EEG samples a volume-conducted, spatially degraded version of the electrical activity, where the potential at any location can be considered a mixture of multiple sources (Makeig et al., 2004). To overcome this limitation, different blind source separation and source reconstruction techniques have been devised. Blind source separation techniques, like independent component analysis (ICA), are increasingly becoming popular both in EEG and in fMRI research, but there are several principal differences in how these techniques are applied in the two domains. In EEG research, temporal ICA (TICA) is usually used, whereas in fMRI research, spatial ICA (SICA) is almost exclusively applied. There are several reasons for this, of which the most important is that the spatial dimension is much larger than the temporal dimension in fMRI data, whereas for EEG data, the temporal dimension is much larger than the number of sources (Calhoun et al., 2001). This methodological difference may impede the direct comparison of EEG and fMRI ICA results. To overcome this obstacle, Knyazev et al. (2011) developed a method, which allows application of SICA to EEG data. A series of simulations showed that both SICA and TICA performed adequately with spatially and temporally independent sources, but SICA outperformed TICA when sources were temporally correlated (Knyazev, 2013b).

The source reconstruction techniques could be roughly divided into two categories: 3D imaging (or distributed) reconstruction methods and equivalent current dipole approaches. The former consider all possible source locations simultaneously, allowing for large and widely spread clusters of activity. The latter rely on a hypothesis that only a few sources are active simultaneously and those sources are focal. It should be emphasized that all EEG source reconstruction methods are probabilistic modeling techniques, which at best point to the most probable location and do not give the “true” localization of sources. Besides, they typically have low spatial resolution. However, it should be kept in mind that fMRI data also represent results of statistical procedures to compare signals between groups or within subjects and do not show the direct structural localization of observed effects.

There potentially are two different approaches to the study of EEG correlates of self-referential processing. Firstly, because simultaneous registration of EEG and fMRI has become possible, the degree of overlap between these two signals in brain regions related to self-referential processing (e.g., the DMN) could be determined. Second and more direct approach would be the study of EEG correlates of self-referential processing *per se*. Below, I will discuss studies, which employed both these approaches and will try to show whether the results correspond, complement, or contradict the existing fMRI framework.

## EEG Correlates of the Default Mode Network

Because DMN mostly operates in a resting state, many simultaneous EEG-fMRI studies attempted to reveal correlations between spontaneous fluctuations of BOLD and cortical electrical activity in this state. Since oscillations constitute the most salient feature of the spontaneous EEG, many of these studies correlated BOLD with different EEG frequency bands. Alpha oscillations have received most attention because they characterize quiet wakefulness and, like DMN, are inversely related to bottom-up sensory processing (Goldman et al., 2002; Laufs et al., 2003a,b; Moosmann et al., 2003; Goncalves et al., 2006; de Munck et al., 2007, 2008; Tyvaert et al., 2008; Jann et al., 2009, 2010; Sadaghiani et al., 2010). The general pattern that has been revealed in these studies is consistent with the picture in which thalamus shows positively correlated activity, while fronto-parietal and occipital regions exhibit negatively correlated activity. Together with studies reporting reduced attention to the external environment, these correlations suggest a reduction of activity in brain regions associated with externally directed attention and a potential increase in activity in the DMN (Larson-Prior et al., 2011). However, there are significant differences in reported positive alpha-band correlations to elements of the DMN (e.g., Laufs et al., 2003b; Ben-Simon et al., 2008; Jann et al., 2009). Laufs (2008) noted that the failure across studies to identify an average cortical BOLD signal pattern, which is positively correlated with alpha power, may be explained by non-uniform brain activity at the population level during periods of prominent alpha oscillations which fMRI group analysis must fail to detect. Later studies, which used more sophisticated approaches to data analysis, tend to show positive correlations of alpha oscillations with the DMN more frequently. Thus, Mantini et al. (2007) incorporated into their analysis EEG bands between 1 and 50 Hz averaged across the entire scalp and correlated with these bands the fMRI time courses of resting-state networks (RSNs) identified by the use of ICA. The DMN and the dorsal attentional network had strong relationship with alpha and beta rhythms, albeit in opposite directions, with the DMN showing positive whereas the attentional network showing negative correlation with these oscillations. Jann et al. (2010) report on the topographic association of EEG spectral fluctuations and RSNs dynamics using EEG covariance mapping. *T*-mapping of the covariance maps indicated that the strongest effects were again in the alpha and beta bands. DMN activity was found to be associated with increased alpha and beta1 band activity. Brookes et al. (2011b) analyzed magnetoencephalographic (MEG) data using a combination of beamformer spatial filtering and ICA. This method resulted in RSNs with significant similarity in their spatial structure compared with RSNs derived independently using fMRI. In this study, the DMN was identified using MEG data filtered into the alpha band. Wu et al. (2010) using parallel ICA decompositions of the fMRI data in the spatial domain and of the EEG data in the spectral domain found widespread alpha hemodynamic responses and high functional connectivity (FC) during eyes-closed rest with predominant negative peaks in occipital, temporal, and frontal regions, biphasic responses in the DMN, and a positive peak in the thalamus. Eyes-open resting abolished many of the hemodynamic responses and markedly decreased FC. On the other hand, Mo et al. (2013) found that visual alpha power was positively correlated with DMN only when the eyes were open. This finding has been interpreted as indicating that under the eyes-open condition, strong DMN activity is associated with reduced visual cortical excitability, which serves to block external visual input from interfering with introspective mental processing mediated by DMN, while weak DMN activity is associated with increased visual cortical excitability, which helps to facilitate stimulus processing. Hlinka et al. (2010) showed that DMN’s FC correlates positively with relative alpha and beta power. Ros et al. (2013) used neurofeedback to reduce alpha rhythm. Compared to sham-feedback, neurofeedback induced an increase of connectivity within regions of the salience network involved in intrinsic alertness and a decrease of connectivity in the DMN. The change in DMN connectivity was positively correlated with changes in “on task” mind wandering as well as resting-state alpha rhythm. Moreover, both mind wandering and alpha change correlated positively with connectivity in clusters of the precuneus both in the neurofeedback and in the sham group. Besides, for the sham group only, a more extensive positive correlation with resting-state alpha change was observed in a region of the MPFC. Hence, both neurofeedback and sham groups remained consistent with the reports of a positive association between alpha synchronization and DMN connectivity (Mantini et al., 2007; Jann et al., 2009; Hlinka et al., 2010). Meyer et al. (2013) investigated the relationship of ICA-derived RSNs and their correlated electrophysiological signals in eyes-open resting state. In 4 of the 12 subjects, negative alpha correlation with visual RSNs was found, however, due to large inter-subject variability, no significant correlations were found on the group level.

Some investigators correlated fMRI BOLD signal with measures of EEG synchronization in the alpha frequency band. Jann et al. (2009) show that the BOLD correlates of global EEG synchronization in the alpha frequency are located in brain areas involved in the DMN. Sadaghiani et al. (2010, 2012) adapted the phase-locking value to assess fluctuations in synchrony that occur over time in ongoing EEG alpha activity. Fluctuations in global synchrony in the upper alpha band correlated positively with activity in several prefrontal and parietal regions, as measured by fMRI. fMRI intrinsic connectivity analysis confirmed that these regions correspond to the well-known fronto-parietal network which has been consistently shown to be recruited by tasks that involve top-down attentional control processes. This apparent disagreement with the Jann et al.’s (2009) study is explained by the fact that different measures of phase synchrony and a fixed vs. individually determined high alpha range are employed in the two studies implying that results might correspond to functionally different oscillations (Sadaghiani et al., 2012). This latter notion is in line with the framework stating that the scalp-recorded alpha is the end-product of many alpha rhythms that are spatially averaged over the scalp (Basar et al., 1997; Nunez et al., 2001). Thus, Ben-Simon et al. (2008) demonstrated two spatially segregated yet simultaneously active networks associated with alpha rhythm modulations, which they call the induced and the spontaneous. These networks might be related to two endogenous processes of the “resting brain,” one, which is tuned outward and is periodic, the other, which is focused inward and is persistent (Ben-Simon et al., 2008). The latter network showed a considerable overlap with the DMN. Two separable alpha-band networks were revealed also in a study by Chen et al. (2013) who employed a four-step analytic approach to the EEG: (1) group ICA to extract independent components; (2) standardized low-resolution tomography analysis (sLORETA) for cortical source localization of IC network nodes; (3) graph theory for FC estimation of epoch-wise IC band power; (4) circumscribing IC similarity measures via hierarchical cluster analysis and multidimensional scaling. During eyes-open compared with eyes-closed condition, graph analyses revealed two salient functional networks with fronto-parietal connectivity: a medial network with nodes in the MPFC/precuneus, which overlaps with the DMN, and a more lateralized network comprising the middle frontal gyrus and inferior parietal lobule. Interestingly, there is a hypothesis that an internal train of thought unrelated to external reality is produced through cooperation between autobiographical information provided by the DMN and the fronto-parietal control network which helps sustain and buffer internal trains of thought against disruption by the external world (Smallwood et al., 2012). This hypothesis explains why activation of the fronto-parietal network and the DMN is often observed together during periods of internally guided thought. If this hypothesis is true, the existence of two separable alpha-band networks associated with the DMN and the fronto-parietal network, respectively, would make functional sense. In any case, the involvement of alpha oscillations in both the top-down attentional control and the integration of internal mental processes are supported by numerous observations (see e.g., Klimesch et al., 2007; Knyazev, 2007 for reviews).

Other EEG frequency bands (most notably theta and gamma) also showed correlations with DMN BOLD signal. Medial frontal theta power changes were negatively correlated with the BOLD response in medial frontal, inferior frontal, p/PCC, inferior parietal, middle temporal cortices, and the cerebellum (Scheeringa et al., 2008). Meltzer et al. (2007) also found that fronto-medial theta most strongly negatively correlates with the MPFC, although negative correlations were also found with other DMN areas such as PCC. In the study by Mizuhara et al. (2004), the frontal midline theta showed negative correlation with BOLD signal over anterior medial regions. The inverse relationship between theta and BOLD in the DMN was also observed in the study by White et al. (2012). There is some evidence that delta, like theta, also shows negative correlation with the DMN. Thus, Hlinka et al. (2010) showed that DMN’s FC correlates negatively with relative delta power. In a study by Dimitriadis et al. (2010), delta activity showed a widespread increase in areas overlapping with the DMN during the performance of arithmetic tasks, which are known to cause DMN’s deactivation. Since delta and theta are indicated as the primary EEG frequencies in limbic structures (i.e., theta in hippocampus and delta in orbito-frontal cortex, see e.g., Knyazev, 2007, 2012 for review) the negative correlations with the DMN may be influenced by projections from these structures to midline frontal regions (e.g., Brazier, 1967, 1968, 1969).

Contrary to theta and delta, gamma (30–50 Hz) power shows positive correlations with DMN BOLD signal at rest (Mantini et al., 2007) and decreases during the transition from resting state to an attention task which is interpreted as a correlate of DMN deactivation (Lachaux et al., 2008; Hayden et al., 2009; Jerbi et al., 2010; Berkovich-Ohana et al., 2012). Moreover, slow changes in the power of gamma oscillations make a significant contribution to the spontaneous local fluctuations of resting-state BOLD signals (Nir et al., 2007, 2008; He et al., 2008; Scholvinck et al., 2010) supporting the notion that gamma processing reflects local neural computations (Canolty and Knight, 2010; Siegel et al., 2012). Most interesting data on gamma-correlates of the DMN have been obtained in studies with depth recordings in humans (e.g., Jerbi et al., 2010). However, Wang et al. (2012) have shown that low-frequency oscillations (<20 Hz), and not gamma activity, predominantly contributed to inter-areal BOLD correlations. The low-frequency oscillations also influence local processing by modulating gamma activity within individual areas (Wang et al., 2012).

Basing on PET and fMRI findings of DMN localization and properties, some investigators attempted to derive EEG correlates of the DMN without simultaneous EEG-fMRI recordings. Chen et al. (2008) compared the spatial distribution and spectral power of seven bands of resting-state EEG activity in eyes-closed and eyes-open condition and termed the defined set of regional and frequency specific activity the EEG-DMN. Fingelkurts and Fingelkurts (2011) used measures of “operational synchrony” of alpha oscillations and found a constellation of operationally synchronized cortical areas including two symmetrical occipito-parieto-temporal and one frontal spatio-temporal patterns (indexed as DMN) that was persistent across all studied experimental conditions. Interestingly, it was further shown, that such DMN operational synchrony was smallest or even absent in patients in vegetative state, intermediate in patients in minimally conscious state, and highest in healthy fully self-conscious subjects (Fingelkurts et al., 2012). Because fMRI research has shown that functional synchrony across elements of the DMN coheres through brain oscillations at very low frequencies (i.e., 0.1 Hz, Fransson, 2005; Fox et al., 2006), some studies investigated very low EEG frequencies (VLF, Vanhatalo et al., 2004; Helps et al., 2008, 2009, 2010; Broyd et al., 2011). It has been shown that VLF has a temporally stable and distinctive spatial distribution across the scalp with maximal power distributed across frontal midline and posterior regions (Helps et al., 2008, 2010). This scalp network shows deactivation of EEG power following the transition from rest to task (Helps et al., 2009, 2010) and these deactivations are correlated with attention performance (Helps et al., 2010; Broyd et al., 2011). Using sLORETA, the sources of this deactivation were localized to medial prefrontal regions, p/PCC, and temporal regions (Broyd et al., 2011). These results suggest similarities between the DMN as identified by fMRI and the VLF EEG network.

Some authors propose that the neural activity at a specific frequency band is unlikely to constitute the electrophysiological correlate of an RSN. Instead, microstates of the EEG signal have been proposed as potential electrophysiological correlates of spontaneous BOLD activity in the DMN (Britz et al., 2010; Musso et al., 2010; Yuan et al., 2012).

In sum, the study of spontaneous EEG correlates of the DMN appear to suggest that low-frequency EEG oscillations of delta and theta bands predominantly at frontal cortical sites correlate negatively with the DMN, whereas higher frequency oscillations (most notably alpha at parietal and occipital regions) show positive correlations with this network. It should be noted that although alpha, beta, and gamma oscillations show positive correlations with the DMN, specificities of these relationships are not equal for the three bands. It appears that alpha (and possibly slow beta) correlates positively with DMN and negatively with attentional networks whereas gamma shows positive correlations with most cognitive processes including attention (e.g., Muller et al., 2000; Fan et al., 2010; Hipp et al., 2011; Ossandón et al., 2012). Very low EEG frequencies could also be considered as promising candidates, although the functional significance of these oscillations has yet to be determined.

## EEG Studies of Self-Referential Processing

All EEG studies of self-referential processing could be subdivided into several categories basing on the nature of EEG phenomena under study and the kind of self-referential processing. Firstly, some studies attempted to correlate spontaneous EEG measures in a resting state with measures of spontaneous self-referential thoughts (e.g., retrospective self-reports). Secondly, EEG correlates of the processing of self-related vs. not self-related external stimuli have been investigated. The latter in turn could be categorized into studies using ERPs or oscillations as the outcome EEG measure. I will describe these three groups of studies separately and will try to summarize how they agree or disagree with each other and the existing fMRI framework.

## Spontaneous EEG Studies

There are few resting-state EEG studies, which attempted to correlate spontaneous EEG measures with measures of self-referential thoughts. Cannon and Baldwin (2012) sought to determine whether the current source density levels in the DMN as measured by sLORETA would correspond to other neuroimaging techniques and to understand the subjective mental activity associated with the DMN during baseline recordings and three experimental conditions. Participants completed subjective reports regarding the mental activities employed during baseline recordings. In all frequency bands from delta to beta, the DMN appeared to be preferentially involved in self-relevant, self-specific, or self-perceptive processes. Knyazev et al. (2011) used a combination of ICA and sLORETA source imaging to reveal RSNs in traditional EEG frequency bands. A short self-report scale was used to measure individual differences in the intensity of self-referential thoughts. Only alpha-band spatial patterns simultaneously showed a considerable overlap with the DMN and a positive correlation with the measure of self-referential thoughts. This group of researchers has replicated their findings in large and diverse groups of subjects coming from two different cultures and found culture-related differences in EEG correlates of self-referential thoughts (Knyazev et al., 2012). Specifically, the self-referential thought-related increase of alpha activity prevailed in the posterior DMN hub in Russian, but in the anterior DMN hub in Taiwanese participants. These culture-related differences could be explained by different self-construal styles that prevail in different cultures (Markus and Kitayama, 1991), but they could be also explained by systematic culture-related differences in personality (see e.g., Gartstein et al., 2005; Knyazev et al., 2008b for the evidence on persistent differences in temperament and personality across the lifespan between Russian and other cultures). This latter explanation seems particularly feasible in view of the evidence that similar differences in EEG correlates of self-referential thoughts have been found between extraverts and introverts (Knyazev, 2013a) and there is ample evidence that Eastern populations in general and Taiwanese population in particular are lower in Extraversion than most more western populations including Russia (see e.g., Allik and McCrae, 2004). This evidence gives interesting hint about the relationship between EEG correlates of self-referential thoughts and the dopaminergic basis of extraversion (Depue and Collins, 1999). Indeed, it has been shown that the association between extraversion and posterior vs. frontal EEG activity is mediated by dopamine (Wacker et al., 2006; Wacker and Gatt, 2010; Koehler et al., 2011) and there is ample evidence that the posterior and the anterior DMN hubs are differentially susceptible to dopaminergic influences (see Knyazev, 2013a for a review of this evidence).

A number of studies investigated EEG correlates of self-related mental processes during meditation. Lehmann et al. (2001) using LORETA images of the EEG gamma frequency band investigated locations of intra-cerebral source gravity centers and showed that self-induced meditational dissolution and reconstitution of the experience of the self involves the right fronto-temporal area. Travis (2001) compared EEG patterns during transcending (described as “silence and full awareness of pure consciousness, where the experiencer is left all by himself” Mahesh, 1963, p. 288, cited from Travis, 2001) to other experiences during Transcendental Meditation practice. To correlate specific meditation experiences with physiological measures, the experimenter rang a bell three times during the session. Subjects categorized their experiences around each bell ring. Transcending, in comparison to “other” experiences, was marked by higher EEG alpha amplitude at parietal sites and higher alpha coherence between Fz and Pz. Travis et al. (2010) showed that, compared to eyes-closed rest, Transcendental Meditation led to higher alpha1 frontal power and lower beta1 and gamma frontal and parietal power, higher frontal and parietal alpha1 interhemispheric coherence and higher frontal and fronto-central beta2 intra-hemispheric coherence. eLORETA analysis identified sources of alpha1 activity in midline cortical regions that overlapped with the DMN. Travis and Shear (2010) summarized that different meditation techniques are associated with different EEG bands. Focused attention techniques are characterized by beta/gamma activity; open monitoring techniques are characterized by theta activity; and self-transcending is characterized by alpha activity. Lastly, Travis et al. (2004) show that oscillatory activity (spontaneous and task-related) correlates with trait-like psychological characteristics along an object-referral/self-referral continuum of self-awareness. Specifically, individuals who described themselves in terms of concrete cognitive and behavioral processes (predominantly object-referral mode) exhibited lower alpha and higher gamma power, whereas individuals who described themselves in terms of an abstract, independent sense-of-self underlying thought (predominantly self-referral mode) exhibited higher alpha and lower gamma power.

Default mode network is one among several networks with different functional properties, including those for orienting attention (Corbetta et al., 2008) and memory encoding and retrieval (Maguire and Frith, 2004; Habecka et al., 2005; Burianova et al., 2010). Whereas task-specific networks are activated when attention is directed toward relevant stimuli, the DMN increases in activity during rest (Buckner et al., 2008). It is still unknown, however, how the brain switches functionally between default and task-specific networks. One interesting hypothesis is that transient functional organization of neural assemblies is brought about by synchronization of neural oscillations (von Stein et al., 2000; Varela et al., 2001; Ward, 2003). It should be borne in mind however that sometimes synchronization of an oscillation within a network may actually reflect the inhibition of this network (see e.g., Klimesch et al., 2007). Several EEG studies compared synchrony and spectral power measures within the task-specific networks (attention and memory) and the DMN during attention/working memory tasks vs. mind wandering. More power and phase synchronization in theta, alpha, and gamma frequency bands has been found during mind wandering between brain regions associated with the DMN, whereas during periods when subjects were focused on performing a visual task, there was significantly more phase synchrony within a task-specific brain network (Kirschner et al., 2012). Increases in theta oscillations in the medial frontal cortex, which are accompanied by decreases in beta and gamma oscillations at the same spatial coordinates and other brain areas, including nodes of the DMN, have been shown during working memory tasks (Brookes et al., 2011a). The increase in frontal theta power during working memory tasks has been shown to correlate with BOLD decrease in regions that together form the DMN (Scheeringa et al., 2009). The same study showed a right posterior alpha power increase, which was functionally related to BOLD decreases in the primary visual cortex and in the posterior part of the right middle temporal gyrus. No correlations were observed between oscillatory EEG phenomena and BOLD in the traditional working memory areas. These findings prompt an assumption that the observed increases in oscillatory power during working memory tasks actually reflect inhibition of neuronal activity that may interfere with working memory maintenance, with theta power increase being related to the inhibition of the DMN while alpha power increase being related to the inhibition of sensory perception (Scheeringa et al., 2009). Children demonstrate a stronger negative correlation between global theta power and the BOLD signal in the DMN during a working memory task relative to adults implying that children suppress this network even more than adults, probably from an increased level of task-preparedness to compensate for not fully mature cognitive functions (Michels et al., 2012). In contrast to power, correlations between instantaneous theta global field synchronization and the BOLD signal were exclusively positive in both adults and children, but only significant in adults in the frontal-parietal and posterior cingulate cortices. Moreover, theta synchronization, in contrast to EEG power, was positively correlated with response accuracy in both age groups. Thus, these studies show that increase of theta power correlates with DMN suppression; increase of theta synchrony correlates with working memory performance; increase of alpha power, on the other hand, correlates with a suppression of sensory networks.

Summing up, the above outlined EEG studies appear to converge in showing that in resting condition, self-related thoughts are accompanied by an increase of spectral power in cortical regions overlapping with the DMN and these changes are most consistently found in the alpha band of frequencies. During working memory tasks, however, the deactivation of the DMN is reflected in an increase of medial frontal theta power with concomitant decrease of beta and gamma oscillations and an increase of alpha power in sensory cortices reflecting inhibition of neuronal activity that may interfere with working memory maintenance.

## EEG Correlates of the Processing of Self-Related Information

Because self-related information could be presented via different sensory and functional domains (e.g., auditory, visual, sensorimotor, verbal, spatial, emotional, and so on), there could be domain-specific and self-specific effects. A meta-analysis by Northoff et al. (2006) of PET and fMRI studies of self-referential processing identified activation in cortical midline structures occurring across all functional domains (e.g., verbal, spatial, emotional, and facial). Cluster and factor analyses indicated functional specialization into ventral, dorsal, and posterior cortical midline areas. The latter encompasses the p/PCC and is considered involved in self-integration (i.e., linkage of self-referential stimuli to the personal context, Northoff and Bermpohl, 2004). It is interesting, therefore, to look how EEG studies corroborate or contradict this framework. I will first present ERP and then oscillation studies of the processing of self-related stimuli.

Own body, own name, and the image of own face are the kind of stimuli that are frequently used in the studies of self-processing. It has been suggested that social cognition is one of the functions of the DMN (e.g., Mitchell, 2006) and it certainly constitutes a part of the self (e.g., Markus and Kitayama, 1991; Han and Northoff, 2009). Therefore, the processing of social stimuli and effects of social and cultural contexts are also relevant to the study of self-referential processing. Because real social behavior (i.e., interactions with other people) is not always possible to organize in a laboratory in a controlled manner, which is needed for EEG registration and subsequent meaningful analysis, virtual (i.e., modeled by means of a computer game) social interactions are frequently used.

## ERP Studies

Many ERP studies of self-referential processing show that the discrimination of self from others is frequently associated with the well-known P300 ERP component, an evoked response to stimuli that are unexpected, salient, or motivationally relevant (Polich and Kok, 1995). Source localization of this response frequently shows activations in DMN structures associated with self-processing. Thus, the own hand elicited a greater positive component (P350–500) than did other hand and the generator of this component was localized in the anterior cingulate cortex (ACC, Su et al., 2010). Mental imagery tasks with respect to the own body have been shown to elicit selective activation of the temporo-parietal junction at 330–400 ms after stimulus onset (Blanke et al., 2005); duration of this activation, but not its strength, were found to correlate positively with perceptual aberration scores (Arzy et al., 2007). A higher P300 wave to the subject’s own face than familiar or unfamiliar faces was observed in several studies (Ninomiya et al., 1998; Scott et al., 2005; Sui et al., 2006). Caharel et al. (2002) did not observe this effect, probably because of the very high occurrence of the subject’s own face, illustrating the major habituation effect of such paradigms. Keyes et al. (2010) observed differences in the ERP waveforms much earlier, with increased N170 and vertex positive potential amplitude over posterior and fronto-central sites, respectively, for self relative to both friend and stranger faces. Cultural difference in neural mechanisms of self-recognition has been investigated both with regard to the long-term cultural experiences (Sui et al., 2009) and after modulation of temporary access to other cultural frameworks using a self-construal priming paradigm (Sui et al., 2013). For British participants, the own-face induced faster responses and a larger negative activity at 280–340 ms (N2) relative to the familiar face, whereas Chinese participants showed reduced N2 amplitude to the own-face compared with the familiar face (Sui et al., 2009). Furthermore, for British participants, priming an interdependent self-construal reduced the default anterior N2 to their own faces. For Chinese participants, however, priming an independent self-construal suppressed the default anterior N2 to their friend’s faces (Sui et al., 2013).

Similarly to the processing of own face, participant’s own name elicits a higher P300 amplitude (e.g., Fischler et al., 1987; Berlad and Pratt, 1995; Muller and Kutas, 1996; Holeckova et al., 2006). By presentation the participant’s first name against a number of other first names in strict equiprobable fashion, it was possible to record an electrophysiological response to the subject’s own name, which is independent of its probability of occurrence (Perrin et al., 1999, 2005). The characteristics of this ERP are consistent with those of the classical P300, but the latency (500 ms) was much longer than that usually obtained in response to pure tones (300 ms), this being probably the consequence of the difference in the length of the stimulus (Perrin et al., 1999). Differential ERPs to the own name were shown in altered states of consciousness, such as sleep (Perrin et al., 1999, 2005; Pratt et al., 1999) and in patients in a vegetative state (Perrin et al., 2006), suggesting that the identification of self-relevant stimuli remains in these states. Using an EEG-PET paradigm, Perrin et al. (2005) have shown that the amplitude of the P300 component, elicited when hearing one’s own name, correlates with regional cerebral blood changes in right superior temporal sulcus, precuneus, and MPFC. Additionally, the latter was more correlated to the P300 obtained for the subject’s name compared to that obtained for other first names. These results are in good agreement with fMRI studies showing differences in activation in MPFC and right paracingulate cortex (Kampe et al., 2003; Staffen et al., 2006) when comparing activation to presentation of the subject’s own name to the activation to presentation of other names. These results are also in good agreement with the proposed critical role of midline structures in self-referential processing (Northoff and Bermpohl, 2004; Lou et al., 2005). Similar effects were observed when the self-relevance effect in object recognition was studied (Miyakoshi et al., 2007).

Effects of the self-relevant possessive pronouns compared to non-self-relevant possessive pronouns were studied in several studies. These studies have shown that self-relevant possessive pronoun elicited significantly larger P300 amplitude than non-self-relevant possessive pronouns (Zhou et al., 2010; Shi et al., 2011) with sources of this activity being identified in MPFC, anterior cingulate, and postcentral cortex (Shi et al., 2011). Walla et al. (2007, 2008) showed that in the time range between 250 and 400 ms the information related to “my” and to “his” could be distinguished over occipital electrodes and in the temporal region. In a study by Esslen et al. (2008), self- vs. other-reference was investigated using trait adjectives in reference to the self or a close friend. The MPFC was found to be more active during the self-reference condition. In an interesting study by Herbert et al. (2011), the effect of emotional valence on ERPs elicited by self-relevant and non-self-relevant pronoun-noun expressions was investigated. From 350 ms onward, processing of self-related unpleasant words elicited larger frontal negativity, whereas processing of pleasant words elicited larger positive amplitudes over parietal electrodes from 450 ms after stimulus onset. This evidence is in line with above discussed evidence linking anterior DMN hub with processing of negative and posterior DMN hub with processing of positive self-related information (Knyazev, 2013a). However, Watson et al. (2007) observed larger N400 amplitudes for words with the self-positivity bias at fronto-central electrode sites. Further research is needed to disentangle the effects of self-reference and emotional valence on cortical electrical responses.

In sum, the discussed ERP studies generally concur with fMRI studies in suggesting that medial cortices (most notably MPFC and ACC) are the crucial structures for processing of self-relevant information. Additionally, they show that the time frame of this processing most frequently coincides with the well-known P300 ERP component.

## Oscillation Studies

Contrary to ERP, which reflects only the evoked (i.e., stimulus-phase-locked) response, oscillations could be spontaneous, induced, or evoked. Spontaneous oscillations as correlates of self-referential processes have been already discussed earlier. This chapter will review studies dealing with induced and evoked responses to self-related stimuli (see earlier in this review a discussion on possible functional meaning of these two kinds of responses). Many of these studies show that alpha suppression appears to be the most salient feature of induced responses to such kind of stimuli. Thus, by means of virtual reality technology, it has been shown that hand ownership and the experience of self-location are reflected in alpha (or mu) band power (8–13 Hz) modulations in bilateral sensorimotor cortices and posterior parietal areas (Lenggenhager et al., 2011; Evans and Blanke, 2013). Electrical neuroimaging showed that alpha power in the MPFC was correlated with the degree of experimentally manipulated self-location (Lenggenhager et al., 2011). Alpha activity in highly similar fronto-parietal regions was also modulated during a motor imagery task (Evans and Blanke, 2013). Hearing subject’s own compared to other names was associated with increased alpha-band desynchronization at frontal sites in time window of 500–1000 ms (Höller et al., 2011). Self-related evaluation on personality traits compared to friend-related evaluation induced stronger desynchronization and decreased phase synchrony in alpha and gamma bands, whereas preparatory self-related attentional orientation was marked by synchronization in these same bands (Mu and Han, 2013). However, in another study, the same authors show that relative to other referential traits, self-referential traits induced event-related synchronization of theta-band activity over the frontal area at 700–800 ms and of alpha-band activity over the central area at 400–600 ms (Mu and Han, 2010).

Several studies investigated EEG correlates of social cognition and behavior. Billeke et al. (2013) used EEG to study the neurobiology of perception of social risk in subjects playing the role of proposers in an iterated ultimatum game. The players were separated to high-risk and low-risk offers. Prior to feedback, high-risk offers generated a drop in alpha activity in an extended network. Moreover, trial-by-trial variation in alpha activity in the medial prefrontal, posterior temporal, and inferior parietal cortex was specifically modulated by risk and, together with theta activity in the prefrontal and PCC, predicted the proposer’s subsequent behavior. Rejections of low-risk offers elicited a higher prefrontal theta activity than rejections of high-risk offers. Using a combination of ICA and sLORETA imaging Knyazev et al. (2011) showed that cortical patterns of alpha desynchronization in response to facial stimuli were different depending on whether these stimuli were presented in a context of social interactions or a judgment of facial affect task. In the former case, alpha desynchronization was found in the posterior DMN hub, whereas in the latter case it appeared at the terminal field of the ventral visual stream. Knyazev et al. (2013) used a computer game to model social interactions with virtual “persons,” which included three major kinds of social behavior: aggressive, friendly, and avoidant. Most salient differences were found between avoidance and approach behaviors, whereas the two kinds of approach behavior (i.e., aggression and friendship) did not differ from each other. Comparative to avoidance, approach behaviors were associated with higher induced responses in most frequency bands, which were mostly observed in cortical areas overlapping with the DMN. The difference between approach- and avoidance-related oscillatory dynamics was more salient in subjects predisposed to approach behaviors (i.e., in aggressive or sociable individuals) and was less pronounced in subjects predisposed to avoidance behavior (i.e., in high trait anxiety scorers). These findings are in line with previous findings showing the effect of these personality traits on the perception of social emotional stimuli (Knyazev et al., 2008a) and oscillatory responses to approach- and avoidance-related cues (Knyazev and Slobodskoj-Plusnin, 2007).

The role of gamma activity in the p/PCC in autobiographical memory retrieval in humans was investigated by means of intracranial recordings (Dastjerdi et al., 2011; Foster et al., 2012). Late-onset (>400 ms) increases in broad high gamma power (70–180 Hz) within p/PCC sub-regions during episodic autobiographical memory retrieval was observed, while it was significantly reduced or absent when subjects retrieved self-referential semantic memories or responded to self-judgment statements, respectively. A significant deactivation of high gamma power was also observed during tasks, which require externally directed attention, such as arithmetic calculation (Foster et al., 2012).

All these studies show that induced oscillatory responses to self-related stimuli are mostly found in cortical areas belonging to the DMN and are most salient in the alpha band of frequencies, although responses in other frequency bands (most notably theta and gamma) are also frequently observed.

Few studies investigated evoked oscillatory responses to self-referential stimuli. Miyakoshi et al. (2010) using the image of participant’s own face observed phase resetting (i.e., evoked response, as measured by ITC values) in the theta band within the medial frontal area during 270–390 ms post-stimulus. Roye et al. (2010) during passive listening observed enhanced evoked oscillatory activity in the 35–75 Hz band for subject’s own telephone ringtone, starting as early as 40 ms after sound onset, and found a co-activation of left auditory areas and left frontal gyri. Active detection of sounds additionally activated the superior parietal lobe supporting the existence of a fronto-parietal network of selective attention. Lastly, Knyazev et al. (2011) observed evoked alpha-band responses to facial stimuli in a social interaction task in the PCC.

## General Summary and Unresolved Questions

It could be summarized that in general, there is a good correspondence between imaging and EEG studies in localizing the self-referential processing in the brain. Across different EEG measures and experimental paradigms, most studies find EEG correlates of these processes within the DMN; most frequently in the MPFC and other midline structures. This is remarkable, because midline structures are not directly accessible from the scalp and their activity could be only modeled by means of source imaging techniques, which have low spatial resolution and well-known other limitations. New information, which comes from EEG research and may not be obtained in fMRI studies concerns the temporal dynamics of self-referential processing and involvement of oscillations. Although some studies find self-processing-related differences in the ERP waveforms (Keyes et al., 2010) or evoked gamma response (Roye et al., 2010) very early (170 and 40 ms, respectively), most other studies show these differences at later stages, which are most frequently associated with the P300 ERP component. Given the well-known functional correlates of this component (i.e., salience detection), this evidence highlights the salience of self-related information and the tendency to pick it out from the stream of external stimuli. Most important and still most disputable question is the relation of EEG oscillations to DMN and self-referential processes. At this stage of our knowledge, it seems prudential not to link these processes to a particular oscillation. Depending on situational context and the kind of self-referential processes, different oscillations may be involved. However, some pattern of their involvement is already discernible. It appears that delta and theta oscillations (most prominently at frontal midline regions) correlate negatively with DMN. Increase of theta power during working memory tasks is related to inhibition of DMN regions (Scheeringa et al., 2009; Brookes et al., 2011a; Michels et al., 2012). Alpha (and possibly beta) oscillations appear to be positively related to DMN and spontaneous self-referential processes and negatively to attentional networks. Alpha also shows most prominent power decrease during processing of external self-related information. The notion of different “alphas” involved in different aspects of attention regulation and top-down processes seems very attractive (Ben-Simon et al., 2008; Sadaghiani et al., 2012; Chen et al., 2013). Gamma oscillations correlate positively with DMN and are definitely involved in self-referential processing, but specificity of their involvement raises doubts because many studies show their involvement in virtually any cognitive process. Finally, oscillations of very low frequencies correlate with DMN, but their involvement in self-related cognitive processes, which typically occur at much faster temporal scales, seems doubtful. I would like to stress that all this relates to spontaneous and induced oscillations. There are too few studies measuring evoked oscillatory responses to self-related stimuli, which make it impossible to derive even preliminary conclusions. Given the above discussed association between self-referential processing and the P300 and existing evidence on crucial role of delta and theta oscillations in shaping this ERP component (see e.g., Knyazev, 2007, 2012 for a review), one would expect that evoked responses in these frequency bands, particularly in the MPFC, should be associated with self-referential processing (see e.g., Miyakoshi et al., 2010). Another very promising field of EEG research, which so far has attracted only limited attention in the study of self-referential processing, is the study of phase relationships between different cortical regions in a frequency band and between different frequencies (Palva and Palva, 2012; Schutter and Knyazev, 2012).

## Conflict of Interest Statement

The authors declare that the research was conducted in the absence of any commercial or financial relationships that could be construed as a potential conflict of interest.
